# Physiological Skin Characteristics of Infants and Children Compared to Those of Women

**DOI:** 10.7759/cureus.19904

**Published:** 2021-11-25

**Authors:** Kaori Zaiki Funamoto, Mariko Akita Furuhashi, Kazuma Muta, Noriyasu Ozawa, Koichi Nakaoji, Kazuhiko Hamada, Katsuko Kikuchi, Hachiro Tagami

**Affiliations:** 1 Research and Development Division, Pias Corporation, Kobe, JPN; 2 Department of Dermatology, Sendai Taihaku Dermatology Clinic, Sendai, JPN; 3 Department of Dermatology, Tohoku University Graduate School of Medicine, Sendai, JPN

**Keywords:** infant, skin physiology, site variation, seasonal variation, children

## Abstract

Introduction

There is little information regarding skin conditions in infants and children, especially with respect to age, anatomical sites, and seasonal variations. This study aimed to compare the physiological skin characteristics of infants and children with those of women.

Methods

This study involved skin measurements and a questionnaire-based survey assessing healthy infants and children aged one month to six years and four months (37 males and 48 females) and 15 healthy women in their twenties in the summer, and healthy infants and children aged two months to six years and seven months (34 males and 45 females) and 15 healthy women in their twenties in the winter. The physiological characteristics of the skin of infants and children were surveyed by age. We excluded infants and children with allergic symptoms at the time of measurement. There were 11 subjects with a history of atopic dermatitis.

Results

Compared with women, infants and children had lower stratum corneum water content and higher transepidermal water loss (TEWL) at most sites. Minimal sebum secretion was observed throughout the body in infants and children aged ≥1 year. The skin surface pH of infants and children was low throughout the body. The questionnaire revealed that skin issues were most common at the anterior neck and cubital fossa, where TEWL was markedly high. These results suggest that barrier function is less developed in the skin of infants and children than in the skin of women.

Conclusions

The physiological characteristics of skin varied depending on age, anatomical site, and season; hence, skincare guidance must be provided according to these factors.

## Introduction

Recently, the living conditions of infants and children have changed substantially, and the incidence of various allergic diseases, including atopic dermatitis, has increased rapidly. Among them, atopic dermatitis, which is partly caused by a decrease in skin barrier function, is worsened by frequent exposure to low-humidity environments [1,2]. Therefore, it is important to apply moisturizer to the skin in such environments. Furthermore, proper skin care in infants and children is associated with the prevention of atopic dermatitis and atopic march [3,4]. An accurate understanding of infant and children skin conditions is important for training and guidance on skin care; however, we have little understanding of the characteristics of infants’ and children’s skin, particularly regarding the variability in skin conditions depending on age, anatomical site, and season. Therefore, to better understand the physiological characteristics of the skin of infants and children in summer and winter, we performed measurements and conducted a survey on skin conditions during these two seasons and compared the findings with those for adult skin. In particular, we selected adult women for comparison because men and women show differences in their physiological skin characteristics [5].

## Materials and methods

Subjects

This cross-sectional study included healthy male and female infants and children (aged one month to six years and seven months) and women in their twenties. The age distribution is shown in Table 1, and the age in months for infants <1-year-old is shown in Table 2.

**Table 1:** Distribution of participants by age and season

Age group (years)	Summer (n)	Winter (n)
0	20	20
1	19	15
2	8	11
3	13	7
4	13	13
5–6	12	13
Total	85	79
Women in their 20s (control group)	15	15

**Table 2:** Distribution of participants <1-year-old by age and season

Age (months)	Summer (n)	Winter (n)
2–3	4	7
4–6	5	6
7–9	7	3
10–11	4	4
Total	20	20

Experimental method

This study was approved by the Ethics Committee of Shibukawa General Hospital (No. 16048-56). The study objectives and content were explained to the parents and guardians of the children, and written consent was obtained.

From 2016-2018, the experiment was conducted during summer (July-September, temperature: 24-30°C, relative humidity: 75-85%) and winter (December-February, temperature: 5-15°C, relative humidity: 40-50%). Before measurements, the staff or parents cleansed the measuring sites using baby wipes. Adult female subjects used facial cleansing sheets and wiped the measuring sites using the same wet wipes as those used for infants and children. Measurements were performed after 15 minutes of acclimatization to a constant temperature and relative humidity (temperature: 23±2°C, humidity: 50±10%) to avoid the effects of perspiration.

Measurement sites

Measurements were taken on the following ten sites: forehead, cheek, anterior neck, inner upper arm, cubital fossa, flexor forearm, abdomen, back, anterior thigh, and anterior lower leg.

Measuring equipment and method

A Corneometer® CM825 (Courage + Khazaka electronic GmbH, Köln, Germany) was used to measure the stratum corneum (SC) water content. Mean values were calculated using five measurements at each site. Transepidermal water loss (TEWL) was measured using a VAPO SCAN® AS-VT100RS (Asch Japan Co., LTD., Tokyo, Japan). Mean values were calculated using two measurements at each site. Skin surface lipids (SSL) were measured using a Sebumeter® SM815 (Courage + Khazaka electronic GmbH, Köln, Germany). Mean values were calculated using two measurements of each site. Skin surface pH was measured one-two times for each site, using a LAQUAtwin® pH meter (HORIBA Ltd., Kyoto, Japan) and dedicated sampling sheets.

Data measurements from each age group were analyzed using Steel’s multiple comparison test. As identical results were observed for each age group, the data of the combined cohort of infants and children aged zero to six years were analyzed using a Mann-Whitney test. All data are presented as the medians, and two-tailed tests were used. Bell Curve for Excel (Social Survey Research Information Co. Ltd., Tokyo, Japan) was used for statistical analyses. All statistical tests were two-sided, and P<0.05 was considered statistically significant.

Questionnaire

The subjects’ parents were requested to complete an original questionnaire reporting the type and site of any skin issues experienced to date and any skincare products currently being used and their frequency of use.

## Results

Stratum corneum (SC) water content

**Figure 1:**
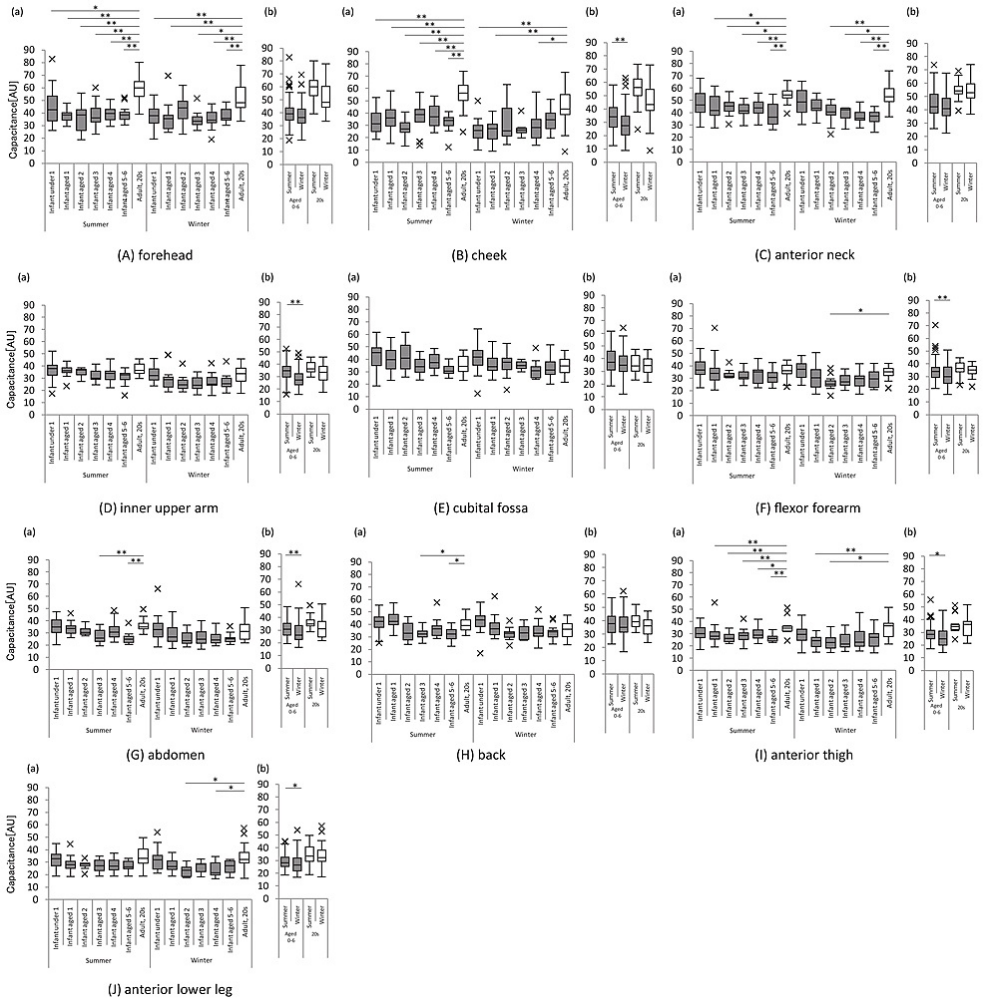
Water content of the stratum corneum The graph on the left shows the water content of the stratum corneum at each site for each age group of infants and children and women in their 20s, in summer and winter (a). The graph on the right shows the water content of the stratum corneum in infants and children and women in their 20s in summer and winter, respectively (b). All analyses were performed using a non-parametric method, and significant differences for the respective age groups are indicated as follows: *: p<0.05 and **: p<0.01. X indicates outliers.

At most sites, the SC water content was lower in infants and children than in women in summer and winter, and this trend was more pronounced in summer than in winter, especially at sites closer to the face (forehead, cheek, and anterior neck). Seasonal variations were noted in the cheek, inner upper arm, flexor forearm, abdomen, anterior thigh, and anterior lower leg of infants and children; the SC water content at these sites was significantly lower in winter than in summer (Figure 1).

Transepidermal water loss (TEWL)

**Figure 2:**
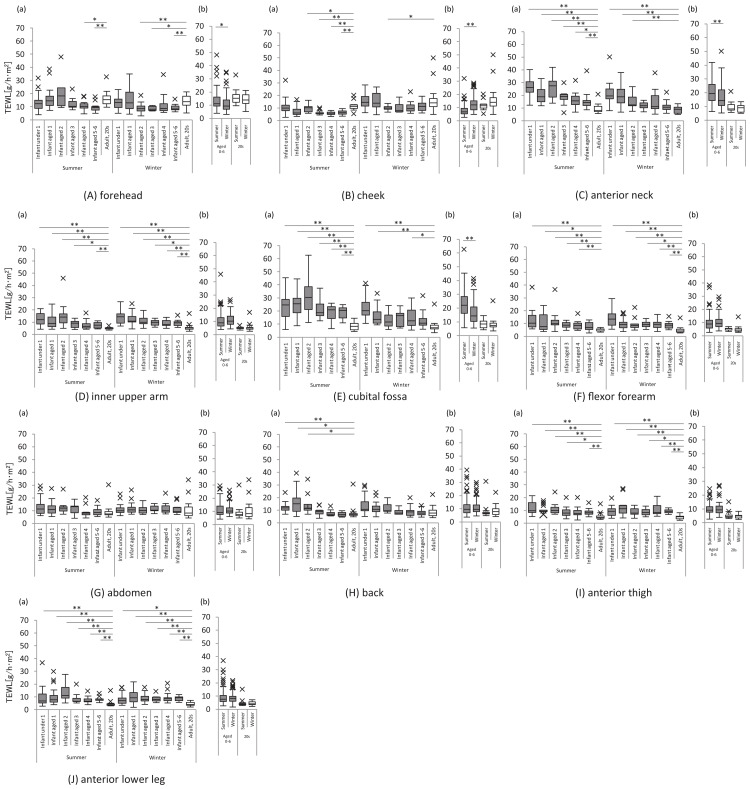
Transepidermal water loss The graph on the left shows the transepidermal water loss at each site for each age group of infants and children and women in their 20s, in summer and winter (a). The graph on the right shows the transepidermal water loss in infants and children and women in their 20s in the summer and winter, respectively (b). All analyses were performed using a non-parametric method, and significant differences for the respective age groups are indicated as follows: *: p<0.05 and **: p<0.01. X indicates outliers.

TEWL at the forehead and cheek was lower in infants and children than in women in summer and winter but was higher at all other sites. TEWL was higher at more sites in infants and younger children, including those aged ≤1 year than in children aged four to six years. TEWL at the anterior neck and back was very high in infants and younger children. Seasonal variation in TEWL was noted in the forehead, cheek, anterior neck, and cubital fossa. TEWL at the cheek was higher in winter than in summer, while that at the forehead, anterior neck, and cubital fossa was higher in summer than in winter (Figure 2).

Skin surface lipids (SSLs)

**Figure 3:**
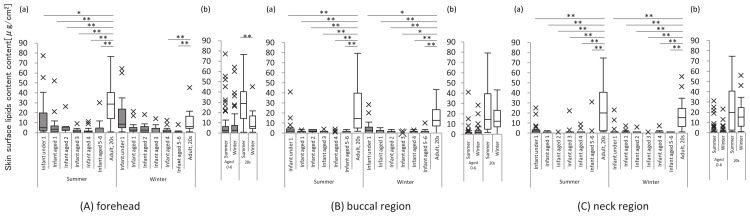
Skin surface lipids The graph on the left shows the skin surface lipid content at each site for each age group of infants and children and women in their 20s, in summer and winter (a). The graph on the right shows the skin surface lipid content in infants and children and women in their 20s in summer and winter, respectively (b). All analyses were performed using a non-parametric method, and significant differences for the respective age groups are indicated as follows: *: p<0.05 and **: p<0.01. X indicates outliers.

In all subjects, SSLs were only found in the forehead, cheek, and anterior neck. SSL levels were lower in infants and children than in women, with negligible SSL levels in children aged >1 year. No seasonal variation existed at any site in infants or children (Figure 3).

Skin surface pH

**Figure 4:**
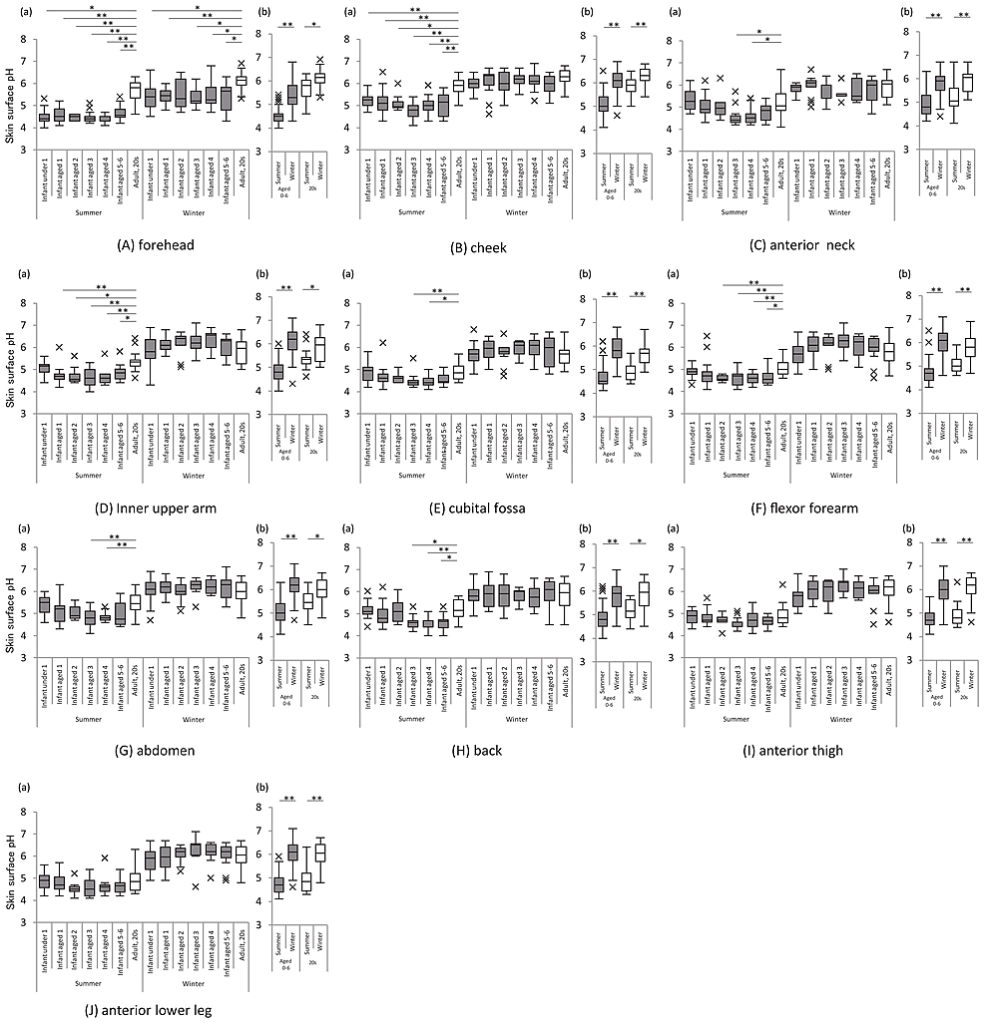
Skin surface pH The graph on the left shows the skin surface pH at each site for each age group of infants and children and women in their 20s, in summer and winter (a). The graph on the right shows the skin surface pH of infants and children and women in their 20s in summer and winter, respectively (b). All analyses were performed using a non-parametric method, and significant differences for the respective age groups are indicated as follows: *: p<0.05 and **: p<0.01. X indicates outliers.

The skin surface pH was lower in infants and children than in women at most sites. The skin surface pH was higher in winter than in summer for all subjects, and maximal seasonal variation was noted in the skin surface pH. Seasonal variation was found at all sites other than the face (forehead and cheek) in all subjects. The skin surface pH of the upper and lower limbs of infants and children increased 1.3-fold from summer to winter (Figure 4).

Questionnaire

The responses to the questionnaire administered at the first visit (N=91) revealed that dryness/roughness (57%), followed by diaper dermatitis (46%), miliaria (42%), infantile seborrheic eczema (40%), and insect bites or bite marks (29%) were the most frequently reported skin problems experienced since birth. Skin problems were most common in the anterior neck and upper limbs, followed by the posterior neck and cheek. Dryness/roughness was most commonly noted on the abdomen and back, followed by the cheek and upper limbs.

When respondents were queried about moisturizer use in summer, 27% answered daily or 5-6 times/week, 15% answered 3-4 times/week, 23% answered 1-2 times/week, and 35% answered less frequent moisturizer use. In contrast, in winter, 55% answered daily or 5-6 times/week, 13% answered 3-4 times/week, 11% answered 1-2 times/week, and 21% answered less frequent moisturizer use. Overall, 15% and 30% of respondents applied moisturizer to the entire body in summer and winter, respectively.

## Discussion

We assessed sites on the face, upper body, and limbs of infants and children presenting with healthy skin and compared the findings with those for healthy adult skin, with respect to skin condition and physiological function in summer and winter. We chose only women for the purpose of comparison with infants and children because the skin of men and women is physiologically different. For example, sebum secretion levels are much higher in men than in women [5]. The SC water content and TEWL results in this study are consistent with previous findings that the SC of infants and children is thin, has low amino acid levels, and, therefore, has immature barrier function [6,7]. The SC water content at the forehead, cheek, and anterior neck was markedly lower in infants and children than in women, and it was high in infants <1-year-old but decreased with age. This may be due to SSLs being unmeasurable in children >1-year-old. Apart from the fact that women have high sebum secretion levels in the face and neck, high SC water content can be maintained with routine skin care. The thinness of the SC may account for facial TEWL being higher than that of the trunk and extremities [8]. TEWL in women was high at the forehead and cheek, which may be due to inflammation in the facial region, an exposed area, or a seborrheic site caused by irritation due to sebum oxidation through UV radiation or external irritation by microorganisms [9].

SSLs are a mixture of sebum secreted from the sebaceous glands with small quantities of SC intercellular lipids, creating a watery, milky skin surface layer that acts as a bacterial barrier and maintains the SC water content. At seborrheic sites, such as the face and head, lipophilic Malassezia may grow, sometimes leading to seborrheic dermatitis. TEWL at the anterior neck and upper and lower limbs was higher in infants and children than in women, and this was more prominent in infants and younger children. This is because intertrigo occurs in the anterior neck and cubital fossa regions; since infants and children have short necks and limbs, they experience more skin friction leading to the deterioration of barrier function. TEWL was higher at the back of infants and younger children, probably because of heat and friction from longer durations of nighttime or afternoon sleep. Cheek TEWL was higher in winter than in summer, similar to that in women [10]. TEWL at the forehead, anterior neck, and cubital fossa of infants and children was higher in summer than in winter. We conducted measurements once the subjects had acclimatized to a constant temperature so that no perspiration occurred. The effect of undetectable perspiration was low, and the differences in TEWL were, therefore, not due to sweat itself. Considering that the neck region and cubital fossa are intertriginous areas, the density of the sweat glands is high; therefore, skin function potentially deteriorates in summer [11]. Heat rash also tended to occur in the neck region and cubital fossa, as reported in the questionnaire, suggesting that skin condition may have deteriorated due to friction.

As a result, SSL levels were unmeasurable at most sites in children >1-year-old. Children secrete minimal sebum before school age but secrete large amounts after puberty [12,13]. Sebum production increases because of androgen activity; thus, there is no secretion in pre-pubescent children. Sebum secretion is observed in infants <1-year-old because of maternal hormones. The previously reported SC water content and TEWL trends [7] may account for the low sebum barrier function and susceptibility to drying out and external irritation of infant skin.

The skin surface pH is reportedly high in healthy newborns and infants and children with atopic dermatitis [7,14]; however, there are few reports on the skin surface pH in healthy infants and children. The differences in endogenous skin flora may account for the low skin surface pH in infants and children. Lactic acid bacteria are endogenous skin flora unique to infants and children, and their metabolites may be responsible for the low skin surface pH [15,16].

TEWL was markedly high at the neck and cubital fossa of infants and children, which are sites that experience many skin issues as reported in the questionnaire. This finding is consistent with previous findings that reductions in barrier function resulted in higher susceptibility to skin issues [17].

Fewer infants and children applied moisturizer daily in summer than in winter, and the same was true for all ages (data not shown). Although barrier function was similarly reduced in summer depending on the site and age, this finding suggests that moisturizers are not applied to infants and children if the skin does not appear dry. In addition, many infants and children did not apply moisturizer to the entire body. Therefore, full-body skin care, regardless of age and season, and careful application of skincare products, without rubbing, should be recommended for high-motion areas, including the anterior neck and cubital fossa, because the skin of infants and children shows poorer barrier function than does the skin of adults. Moreover, people should be informed that the facial skin (forehead and cheek) of infants and children has a lower SC water content than adult skin and dries out more easily. Skincare should be focused on the face, abdomen, and back, as these areas often experience dryness/roughness. Nevertheless, studies with larger sample sizes and more age groups distributed over a larger geographic area are warranted.

## Conclusions

The physiological skin characteristics of infants and children varied depending on age, anatomical site, and season. This study provided valuable data related to skin physiology in healthy infants and children, which remains underreported. Our study results indicated that the most appropriate skincare for infants and children should be chosen based on age, anatomical site, and season to prevent further reduction of skin barrier function, which is considered a trigger for allergic march.
